# Integrated analysis of DNA methylation and microRNA regulation of the lung adenocarcinoma transcriptome

**DOI:** 10.3892/or.2015.4023

**Published:** 2015-05-29

**Authors:** JIANG DU, LIN ZHANG

**Affiliations:** Department of Thoracic Surgery, Chinese Medical University Affiliated No. 1 Hospital, Shenyang, Liaoning 110001, P.R. China

**Keywords:** lung adenocarcinoma, differentially expressed genes, microRNA, transcription factors, methylation

## Abstract

Lung adenocarcinoma, as a common type of non-small cell lung cancer (40%), poses a significant threat to public health worldwide. The present study aimed to determine the transcriptional regulatory mechanisms in lung adenocarcinoma. Illumina sequence data GSE 37764 including expression profiling, methylation profiling and non-coding RNA profiling of 6 never-smoker Korean female patients with non-small cell lung adenocarcinoma were obtained from the Gene Expression Omnibus (GEO) database. Differentially methylated genes, differentially expressed genes (DEGs) and differentially expressed microRNAs (miRNAs) between normal and tumor tissues of the same patients were screened with tools in R. Functional enrichment analysis of a variety of differential genes was performed. DEG-specific methylation and transcription factors (TFs) were analyzed with ENCODE ChIP-seq. The integrated regulatory network of DEGs, TFs and miRNAs was constructed. Several overlapping DEGs, such as v-ets avian erythroblastosis virus E26 oncogene homolog (*ERG*) were screened. DEGs were centrally modified by histones of trimethylation of lysine 27 on histone H3 (H3K27me3) and di-acetylation of lysine 12 or 20 on histone H2 (H2BK12/20AC). Upstream TFs of DEGs were enriched in different ChIP-seq clusters, such as glucocorticoid receptors (GRs). Two miRNAs (miR-126-3p and miR-30c-2-3p) and three TFs including homeobox A5 (HOXA5), Meis homeobox 1 (MEIS1) and T-box 5 (TBX5), played important roles in the integrated regulatory network conjointly. These DEGs, and DEG-related histone modifications, TFs and miRNAs may be important in the pathogenesis of lung adenocarcinoma. The present results may indicate directions for the next step in the study of the further elucidation and targeted prevention of lung adenocarcinoma.

## Introduction

Lung cancer is presently the leading cause of global cancer-related death, with an increasing prevalence and mortality. Smoking is the predominant risk factor for lung cancer. While, in East Asia, ~30% of patients suffering from lung cancer were never smokers (1,2), and non-smoking-related lung cancer can also occur in current and former smokers (3). Unfortunately, lung cancer has not been solved in regards to prevention or diagnosis or treatment.

In addition to small cell lung cancer (SCLC) accounting for 10–15% of lung cancer cases, non-small cell lung cancer (NSCLC) represents ~85–90% of overall lung cancer cases (4). NSCLC is also subdivided into three histologic types, including adenocarcinoma, squamous cell carcinoma and large cell undifferentiated carcinoma. Lung adenocarcinoma accounts for almost 40% of NSCLC. Due to the relatively high incidence of lung adenocarcinoma, much research has been conducted to elucidate its nature and mechanisms.

Previous studies have found various genes related to lung adenocarcinoma. Su *et al* (5) found that a higher level of cyclo-oxygenase-2 decreased the survival rate of patients through many mechanisms, such as a corresponding higher level of vascular endothelial growth factor that stimulated the growth and migration of cancer cells (6), a higher lymphatic vessel density that reduced the restriction of cancer cell invasion (7), and enhanced lymph node metastasis that accelerated the metastasis of cancer cells (8). Mutations of oncogene *K-ras* and tumor-suppressor gene *TP53* have a strong link with lung adenocarcinoma (9). Other fusion genes have been further studied concerning the correlation with lung adenocarcinoma. Fusion of the kinesin family member 5B and *RET* proto-oncogene was found to occur in a subset of NSCLC (10). Fusion genes of echinoderm microtubule associated protein like 4 - anaplastic lymphoma receptor tyrosine kinase and kinesin light chain 1 - anaplastic lymphoma receptor tyrosine kinase were also found in lung adenocarcinoma (11).

To date, the pathogenesis of NSCLC and lung adenocar-cinoma is difficult to determine. To reduce the enormous morbidity and mortality of lung adenocarcinoma, it is critical to identify lung adenocarcinoma-associated genes and mechanisms. Integrated analysis of full DEGs and the expression of regulatory factors such as methylation, mRNA splicing, transcription factors (TFs) and microRNAs (miRNAs) is an effective method for disease pathogenesis study. In the present study, DEGs, exons and isoforms, as well as DEG-related methylation, TFs and miRNAs were integrated and analyzed.

## Materials and methods

### Datasets

The raw experimental data under accession no. GSE 37764 (12) used in the present study are publically available in the Gene Expression Omnibus (GEO) database (http://www.ncbi.nlm.nih.gov/geo). These data, which include expression profiling, methylation profiling and non-coding RNA profiling of 6 never-smoker Korean female patients, were produced by high throughput sequencing. The histologic origins were cancer tissues and adjacent normal tissues of non-small cell lung adenocarcinoma. In the present study, using normal tissues as control, the molecular variations in tumor tissues were identified. The platform of this data is GPL10999 (Illumina Genome Analyzer IIx, *Homo sapiens*).

### Methylation profiling and differentially methylated gene screening

Trimmomatic (13) software package, a flexible, pair-aware and efficient preprocessing tool for Illumina sequence data, is often used to remove low quality reads and trim adaptor sequences. In the present study, the methylated DNA immunoprecipitation-sequencing (MeDIP-seq) was preprocessed with the Trimmomatic (13). During the preprocessing of the Illumina reads, a minimum quality cutoff on the first and last bases using LEADING: 3 (trim the leading nucleotides until quality >3) and TRAILING: 3 (trim the trailing nucleotides until quality >3) was imposed, and a minimum sliding window quality using SLIDINGWINDOW: 4:15 (trim the window of size four for reads with local quality below a score of 15) was subjected. In addition, the resulting reads shorter than 25 bases were discarded. Then the Bowtie (14) alignment algorithm (with default parameters) was used to align the Illumina reads to the human reference genome (hg19), and SAM tools (15) was applied to remove PCR duplicates. The differentially methylated regions (DMRs) were identified by MEDIPS (16) in R. with false discovery rate (FDR) <0.1. Each DMR contains multiple methylated loci, and the determination of overlaps between methylation loci and the adjacent genes were computed using the BED Tools (17) software. Briefly, differentially methylated loci between −2,000 and +1,000 bp around transcription start site (TSS) were selected, and the adjacent genes were defined as differentially methylated genes (DMGs).

### Gene expression profile analysis

RNA-seq reads were cleaned to remove low quality regions and sequencing adaptors utilizing Trimmomatic (13) software package (LEADING: 3, TRAILING: 3, SLIDINGWINDOW: 4:15, MINLEN: 36). These massively parallel short reads were subsequently mapped to a reference genome with TopHat (18) (no >5 bases mismatch). Since multiexon genes can encode different transcripts and multiple transcript variants encode different isoforms, differentially expressed exons were analyzed by DEXSeq (19) in R. and differential expression analysis of genes and transcript isoforms were performed with Cufflinks (20) algorithm. The parameters of DEXSeq and Cufflinks were default values. The thresholds were q-value <0.05 and fold-change (FC) >2. Comparing the cancer tissues and control, genes and exons with average expression levels >10 FPKM (fragments/kilobase of transcript/million mapped reads) were defined as differentially expressed.

### Screening of differentially expressed miRNAs

High-throughput sequencing data for miRNA expression were cleaned to remove low quality regions and sequencing adaptors utilizing Trimmomatic [2] software package (LEADING: 3, TRAILING: 3, SLIDINGWINDOW: 4:15, MINLEN: 25). Reads of millions of miRNA sequences were aligned to the genome and the expression value of each miRNA was measured using miRExpress (21). Subsequently, the screening of differentially expressed miRNAs was performed with DESeq2 (22) in R/Bioconductor FDR <0.01; FC >2; base mean >10). Conserved miRNA targets were retrieved from public websites (TargetScan, http://targetscan.org) (23).

### Functional analysis of a variety of differential genes

Gene Ontology (GO) functional enrichment and annotation of differential genes, including differentially methylated genes and DEGs, were computed using the database for annotation, visualization and integration discovery (DAvID) (24). The annotation of miRNA-target DEGs was performed with TarBase 6.0 database (capturing the exponential growth of miRNA targets with experimental support) (25). Then the DIANA miRPath v.2.0 (26) was used to determine molecular pathways potentially altered by differentially expressed miRNAs based on the Kyoto Encyclopedia of Genes and Genomes (KEGG) database. The ChIP-X Enrichment Analysis (ChEA) and the ENCODE ChIP-seq (28) were utilized to search for enriched TFs located upstream of the DEGs. TF-target genes were predicted and combined with differentially expressed miRNAs and DEGs using mirConnX (29) with Pearson’s correlation coefficient >0.96 and then, the integrated network of TFs, miRNAs and TF-target DEGs were constructed and analyzed.

## Results

### Differentially methylated regions and genes

**Figure 1:**
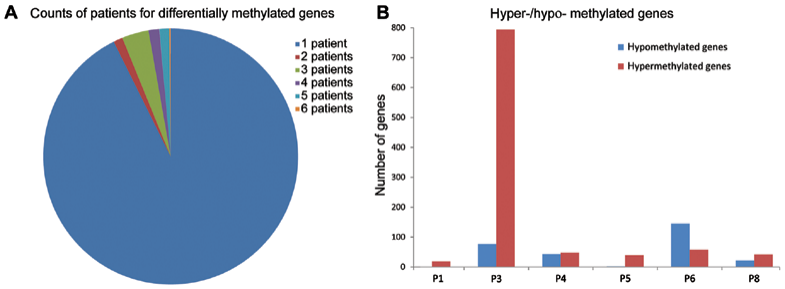
Differentially methylated genes. (A) Gene frequency of methylation in 6 patients. (B) Number of methylated genes in 6 patients. Red column indicates hypermethylated genes and blue column indicates hypomethylated genes. The x-coordinate represents 6 patients and the y-coordinate represents the gene number.

After comparison of the MeDIP-Seq data between cancer tissues and para-carcinoma tissues of 6 non-small cell lung carcinoma patients, DMRs and DMGs were obtained. Most of the DMGs (>90%) were detected in one patient only (Fig. 1A). Only 82 genes were found in 2 or more patients and ~1/3 of the DMGs (34) were located in the mitochondrial genome. The numbers of DMGs in 5 patients were similar except in 1 patient (P3) (Fig. 1B). In patient P3, the hypermethylated genes were significantly more than in the others.

**Figure 2:**
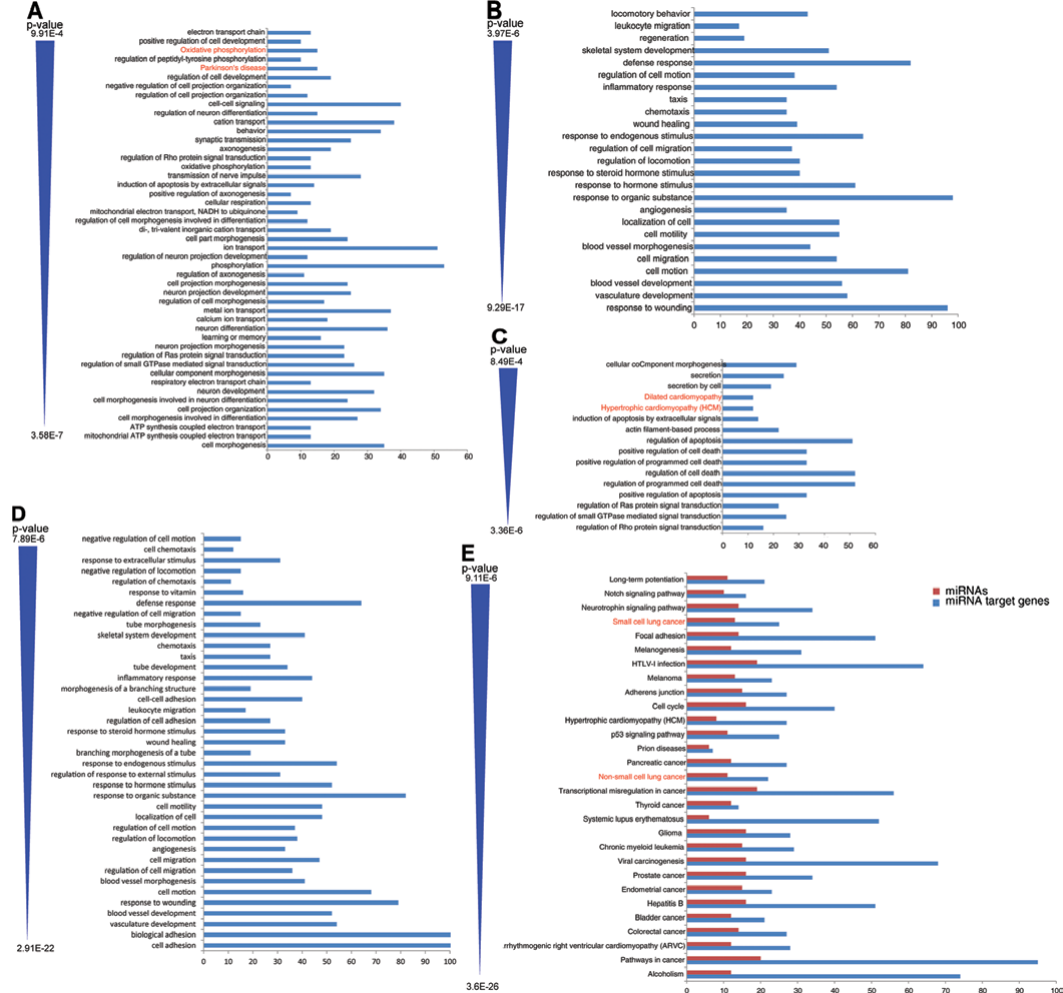
Functional annotations of the differential genes. (A) GO and KEGG analysis of the DMGs. Red indicates the KEGG pathways. (B) GO analysis of the DEGs. Red indicates KEGG pathways. (C) GO and KEGG analysis of differentially expressed exon-related genes. (D) GO analysis of differentially expressed isoform-related genes. (E) GO and KEGG analysis of differentially expressed microRNAs. The x-coordinate represents p-value and the y-coordinate represents gene number.

Functional GO analysis showed that the DMGs were mostly associated with metabolic pathways (Fig. 2A). The most commonly enriched GO terms were cell morphogenesis, mitochondrial ATP synthesis coupled electron transport and ATP synthesis coupled electron transport.

KEGG pathway analysis identified two enriched pathways, respectively: Parkinson’s disease (hsa: 05012) and oxidative phosphorylation (hsa: 00190) (Fig. 2A).

### Gene expression profile analysis

DEG screening analysis found that a total of 1,498 genes were differentially expressed between the cancer tissues and adjacent normal tissues, and 1207 isoforms of 1,103 genes were differentially expressed. Additionally, 1,286 exons of 916 genes were also differentially expressed.

Functional GO analysis showed that most of these differential genes were related to cell migration and apoptosis (Fig. 2B-D). The most commonly enriched terms of genes were response to wounding, vasculature development and blood vessel development. The most commonly enriched terms of the exons were regulation of Rho protein signal transduction, regulation of small GTPasse mediated signal transduction and regulation of Ras protein signal transduction. The most commonly enriched terms of transcripts were cell adhesion, biological adhesion and vasculature development.

KEGG pathway analysis of the differentially expressed exons found two enriched pathways named hypertrophic cardiomyopathy (HCM) (hsa: 05410) and dilated cardiomyopathy (hsa: 05414) (Fig. 2C).

**Figure 3:**
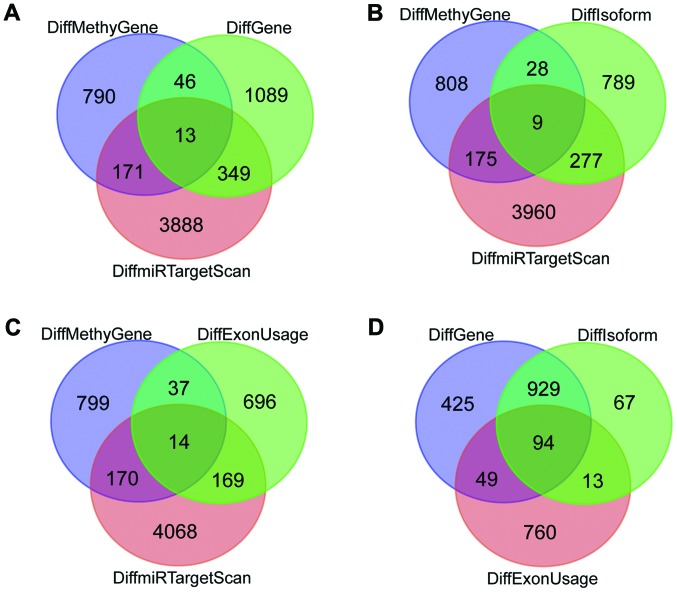
Comparison of DMGs, differentially expressed transcripts and differentially expressed microRNA-target genes. (A) Comparison of DMGs, DEGs and differentially expressed miRNA-target genes. (B) Comparison of DMGs, differentially expressed isoform-related genes and differentially expressed miRNA-target genes. (C) Comparison of DMGs, differentially expressed exon-related genes and differentially expressed miRNA-target genes. (D) DEGs, differentiall expressed isoform-related genes and differentially expressed exon-related genes.

**Figure 4:**
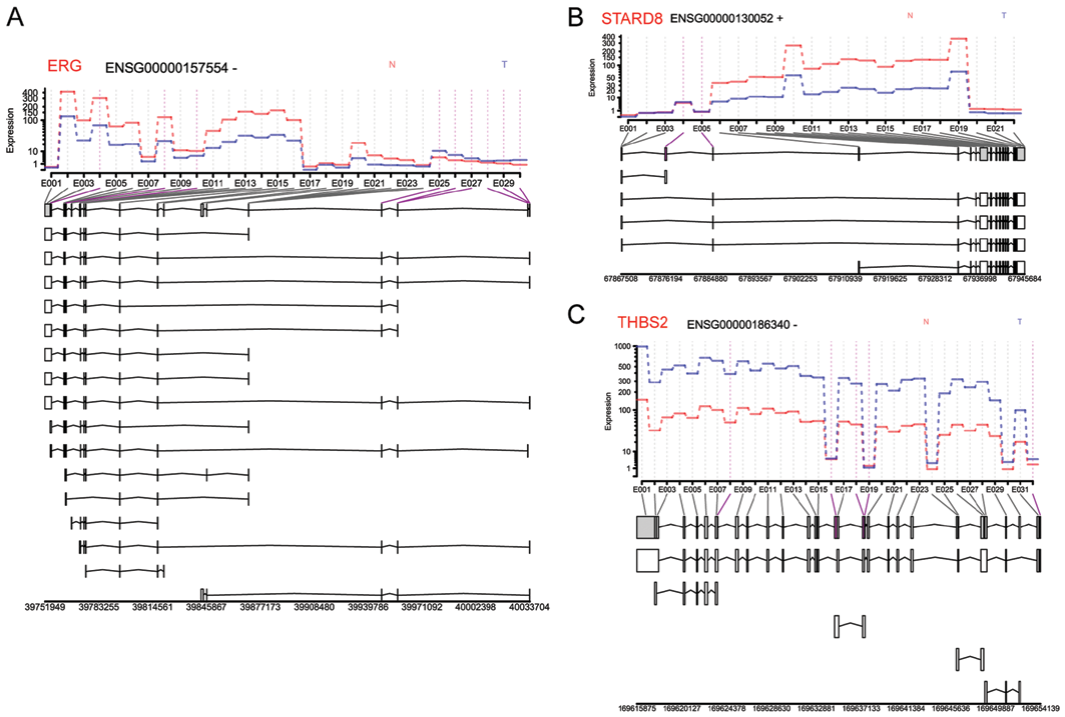
(A–C) Alternative splicing of three overlapping genes. The x-coordinate represents the location on the chromosome and the y-coordinate represents the gene expression level.

A total of 541 genes among these differential genes possessed only 1 differentially expressed exon. As shown in Figs. 3 and 4, the expression levels of different exons corresponding to the same one gene had dozens of time variations. Several exons with a lower expression level appeared almost unanimously between the cases and control, while others displayed a significant difference between these two groups.

There were 94 common genes differentially expressed at the levels of genes, isoforms and exons (Fig. 3D).

### Differential expression of miRNAs

Within 50 precursors, 44 miRNAs were detected as differentially expressed miRNAs. The most overexpressed miRNAs were miR-6510, miR-301b and miR-183. miR-144, miR-486 and miR-451a were the most commonly downregulated miRNAs.

GO and pathway analysis shown that the differentially expressed miRNAs were correlated with many disease-related pathways (Fig. 2E). The top three enriched pathways, respectively, were alcoholism (hsa: 05034), pathways in cancer (hsa: 05200) and arrhymogenic right ventricular cardimyopathy (ARvC) (hsa: 05412). NSCLC (hsa: 05223) and small cell lung cancer (hsa: 05222) were also enriched pathways.

Nine differentially expressed miRNA-target genes with methylation were differentially expressed at levels of isoforms (Fig. 3B) and 14 at the level of exons (Fig. 3C).

### Integrated analysis of the differential genes

Thirteen differentially expressed miRNA-target genes included differentially methylated genes, and also differentially expressed exon-related genes. Furthermore, they were regulated by differentially expressed miRNAs (Table I, Fig. 3A). Yet, differential methylation of these 13 genes was observed only in one patient. In addition, ribosomal protein S kinase, 90 kDa, polypeptide 2 (*RPS6KA2*), DOT1-like histone H3K79 methyltransferase (*DOT1L*) and thrombospondin 2 (*THBS2*), included in these 13 genes, were detected with both hypomethylation and hypermethylation.

The overlapping genes among the differentially methylated genes, DEGs and isoforms, differentially expressed exon-related genes, differentially expressed miRNA-targeted genes were v-ets avian erythroblastosis virus E26 Oncogene Homolog (*ERG*), StAR-related lipid transfer domain containing 8 (*STARD8*) and *THBS2*. Isoforms and expression levels of these 3 genes are shown in Fig. 4

**Figure 5:**
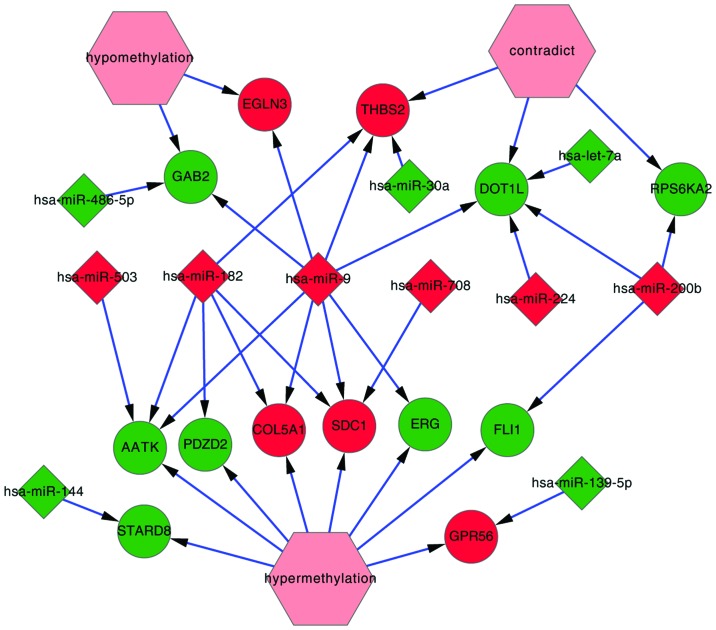
Methylation and microRNA regulatory network of 13 DEGs. Downregulated and upregulated genes are shown in green and red circles, respectively; overexpressed and downregulated miRNAs are displayed in red and green rhombuses, respectively. DEGs, differentially expressed genes.

The possible regulatory networks of methylation, miRNA expression and gene expression are shown in Fig. 5. There were 6 overexpressed and 5 downregulated miRNAs, 5 upregulated and 8 downregulated genes, 2 genes with hypomethylation, 8 genes with hypermethylation and 3 genes with contradiction. In the 11 miRNAs, miR-9 (degree= 8) and miR-182 (degree=5) had more degrees than the others. Genes including *DOT1L*, apoptosis-associated tyrosine kinase (*AATK*), syndecan 1 (*SDC1*) and *THBS2* had more degrees.

### Transcription analysis of DEGs

**Figure 6:**
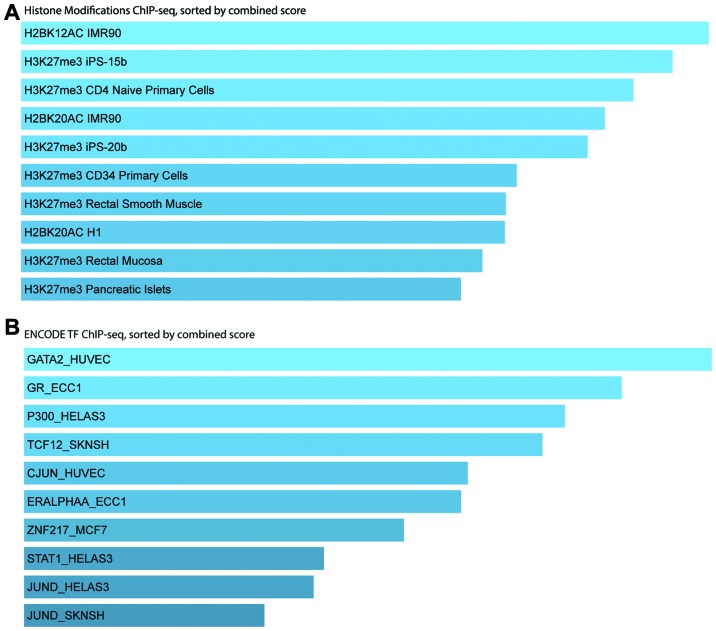
Enrichment analyses of (A) histone modifications and (B) upstream transcription factors of the differentially expressed genes.

ChEA2 analysis results indicated that the screened DEGs were modified and regulated by multi-cancer cell line histones including tri-methylation of lysine 27 on histone H3 (H3K27me3) and di-acetylation of lysine 12 or 20 on histone H2 (H2BK12/20AC), which pertained to the ENCODE database (Fig. 6A). The upstream TF binding patterns were not as clustered as that of the histone modification; they were enriched in different ChIP-seq clusters of TFs in different cell lines (Fig. 6B), such as GATA2 and CJUN in human umbilical vein endothelial cells (HUvECs), glucocorticoid receptors (GRs) and estrogen receptor (ER)α in endometrial cells (ECC1), while, P300, signal transducer and activation of transcription (STAT1) and JUND in HeLaS3 cells.

**Figure 7:**
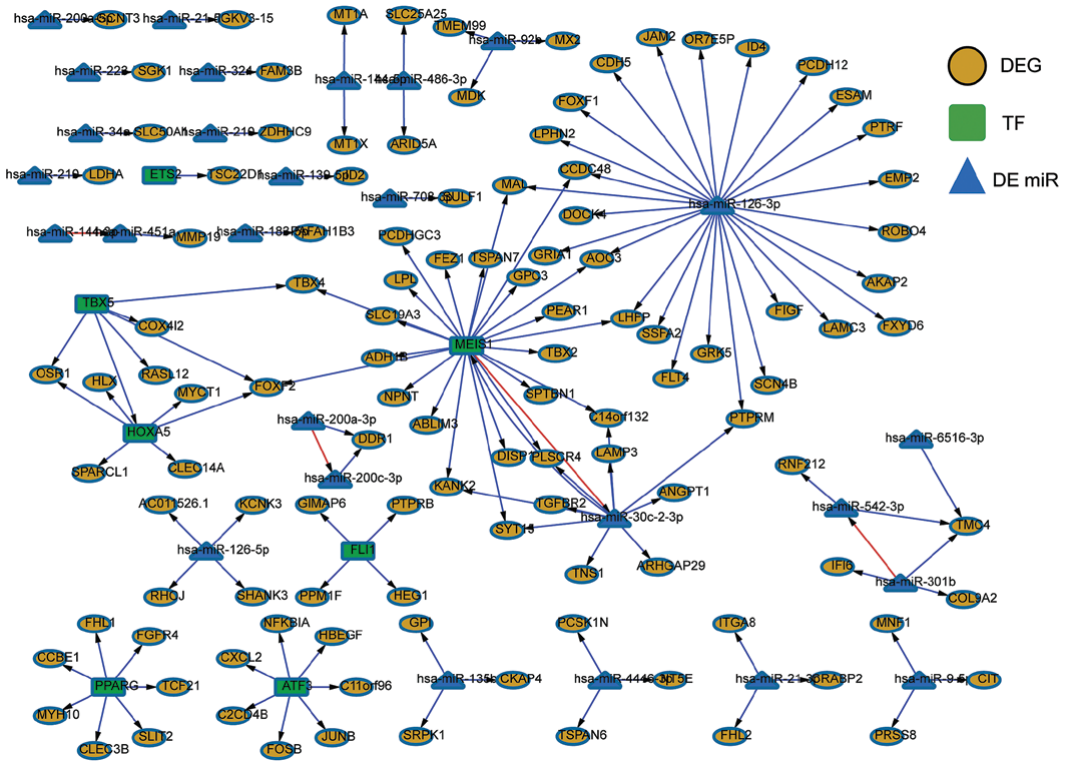
Transcription factor-microRNA regulatory network of the differentially expressed genes. Differentially expressed genes, transcript factors and differentially expressed miRNAs are shown in yellow, green and blue, respectively.

DEGs were regulated by TFs and miRNAs, and the regulatory network was constructed as shown in Fig. 7. There were 116 DEGs, 72 TFs and 7 differentially expressed miRNAs. miR-126-3p served as a ‘hub’ in the gene regulatory network which regulated 26 DEGs. The TF MEIS1 was another ‘hub’, which also regulated 22 DEGs and miR-30c-2-3p. Several sub-networks with homeobox A5 (HOXA5), Meis homeobox 1 (MEIS1), T-box 5 (TBX5), miR-126-3p and miR-30c-2-3p as centers shared several nodes and then formed another greater regulatory network. The remaining sub-networks were detached from each other.

## Discussion

NSCLC accounts for ~85% of all lung cancer cases (30) and remains the leading cause of cancer-related death worldwide (31). Lung adenocarcinoma, the major subtype of NSCLC responsible for more than 500,000 mortalities/year worldwide (32), is associated with a poor prognosis. In the present study, differentially methylated regions, differentially expressed miRNAs and transcriptomics between different tissues from 6 non-small cell lung adenocarcinoma patients were analyzed. Several DEGs, miRNAs and TFs were screened, which were expected to be associated with metabolism, cell apoptosis or various diseases; thus, they may be important in the progression of lung adenocarcinoma. Kim *et al* (12) identified various novel genetic aberrations, gene network modules and miRNA-target interactions within the same dataset, yet, it is distinct from ours. In addition, the pathogenesis of lung adenocarcinoma is far from clear. With the different bioinformatics tools, the results of the same analysis were slightly different from those of Kim *et al* (12). The new information obtained from the present study may help to illuminate the molecular mechanisms of this disease.

The methylation analysis results of the 6 patients were relatively diverse. Since a relatively large number of methylation sites are concentrated in the mitochondrial genome, it is diffi-cult to research the connections between methylation and gene expression. Additionally, *RPS6KA2*, *DOT1L* and *THBS2* were detected with both hypomethylation and hypermethylation. This may be relatively related to the great individual differences of the methylated regions in the patients. However, previous research has confirmed that DNA methylation is critical in lung cancer (33). Combined with the subsequent analysis of differential expression of genes, isoforms and exons, screening of miRNA-related genes, three overlapping genes were obtained. ERG, a member of the *ETS* oncogene family (34), is intimately involved in the development of multiple cancers including prostate cancer (35). The *TMPRSS2-ERG* gene fusion is now a specific biomarker of prostate cancer (36). There is little research on ERG and lung adenocarcinoma. As cancer develops from glands which are the same as prostate cancer, ERG may also be related with lung adenocarcinoma. *STARD8* was found downregulated and highly methylated in the present study. Durkin *et al* (37) suggested that *STARD8* is a tumor-suppressor gene encoding DLC-3 to suppress tumor cell growth. It has a higher level of methylation in colorectal cancer than other types of cancer (38). *THBS2*, an upregulated gene, encodes a protein belonging to the thrombospondin family. This protein has been shown to function as a potent inhibitor of tumor growth and angiogenesis, and it may be involved in cell adhesion and migration (39). *THBS2* also has CpG island methylation in malignant ovarian tumors (40). Therefore, STARD8 and THBS2 may also be involved in lung adenocarcinoma.

Gene expression is under the elaborate control of interrelated factors including TFs and histone modification. In the present study, comparative analysis of histone modifications in tumor and normal tissues was conducted. This revealed that DEGs were centrally regulated by H3K27me3 and H2BK12/20AC in several cancer cell lines. H3K27me3 is regarded as related to gene silencing (41). The H3K27me3 marker is associated with promoters of all hypermethylated genes associated with tumor suppressors in cancer cells (42). With the prevalent regulation executed by H3K27me3 on DEGs screened in the present study, abnormal modification of H3K27me3 may play an important role in lung adenocarcinoma. Studies have shown that high expression of histone H3K27me3 is related with a good prognosis of patients with NSCLC; namely, the higher expression of histone H3K27me3, the better the prognosis of patients (43). Functional analysis of DEGs and isoforms revealed that they were enriched in the process of hormonal responses. Enrichment analysis of ChIP-seq presented that DEGs were enriched in different ChIP-seq clusters including GR and ERα of TFs. This indicated that regulation of ER and GR may be associated with lung adenocarcinoma. Studies have shown that ERα and ERβ, especially ERβ, are expressed in NSCLC to induce tumor cell proliferation (44). It was found that midkine plays a pivotal role in epithelial-mesenchymal transition in lung adenocarcinoma (45). Enhanced ERβ-mediated estradiol dysregulates midkine expression (46). In previous research, GR, a member of the nuclear hormone receptor family, mediated cancer cell apoptosis and thereby slowed tumor growth (47). GR is down-regulated by increased promoter methylation, which is similar to mechanisms associated with common tumor-suppressor genes (48).

The constructed TF and miRNA regulatory networks showed that hub nodes including miR-126-3p, miR-30c-2-3p, HOXA5, MEIS1 and TBX5 were markedly different in two separate TF and miRNA enrichment analyses, and their levels were significantly decreased in cancer tissues. As crucial upstream genes, their significant change in the expression level may affect a plurality of downstream target genes. Expression of most of the HOX family member are altered in NSCLC cells significantly (49). Contradictory results were found by Abe *et al* (49) who detected the downregulation of HOXA5 in NSCLC. Whether HOXA5 regulates various lung cancer-related genes or what changes it undergoes in lung adenocarcinoma, remains to be elucidated. The relationships of two other TFs including MEIS1 and TBX5 with lung cancer are unclear. It is known that MEIS1 is one of the co-factors of the HOX family (especially for HOXA7 and HOXA9) (50). Together, they are involved in human leukemogenesis (51). TBX5 regulates cell proliferation during cardiogenesis (52) and it is related to cell migration as well as cell proliferation in cancers (53). miR-126 has a clearer relationship with NSCLC and could inhibit the proliferation and invasion of NSCLC cells (54). Downregulation of miR-30c promotes cell migration and invasion of NSCLC cells (55). Despite the fact that altered expression of these two miRNAs has long been known, the exact regulatory mechanisms remain to be studied. The present integrated analysis found that possible target expression levels of these two miRNAs may also undergo significant changes. This indicates one direction for further study.

Differentially methylated regions, differentially expressed miRNAs and transcriptomics of normal and cancer tissues were analyzed. Three possible lung adenocarcinoma-related DEGs including *ERG*, *STARD8* and *THBS2* were identified. Moreover, DEG-related histone modifications and TFs were screened and underwent integrated analysis. Lung adenocar-cinoma-related DEGs may be under comparable regulation of histones. Moreover, several TFs and miRNAs may play critical roles in the tumorigenesis of lung adenocarcinoma. These results provide the foundation for further lung adenocarci-noma research, and these results must be confirmed through additional experiments.

## Figures and Tables

**Table I:** Thirteen differentially expressed genes under the regulation of differentially expressed miRNAs and methylation.

Name	miRNA	Expression	N	T	log2FC (T/N)	Hyper-methylated sample
ERG	hsa-miR-9	1	30.2622	7.6856	−1.97729	1T
FLI1	hsa-miR-200b	1	35.8052	11.0298	−1.69876	1T
RPS6KA2	hsa-miR-200b	1	39.4554	19.498	−1.01689	1N1T
STARD8	hsa-miR-144	−1	15.475	4.3143	−1.84274	1T
PDZD2	hsa-miR-182	1	17.9635	5.28059	−1.76629	1T
GPR56	hsa-miR-139-5p	−1	32.3344	65.4073	1.01638	1T
SDC1	hsa-miR-182	1	67.1969	206.511	1.61975	1T
SDC1	hsa-miR-708	1	67.1969	206.511	1.61975	1T
SDC1	hsa-miR-9	1	67.1969	206.511	1.61975	1T
EGLN3	hsa-miR-9	1	5.38757	30.8381	2.51701	1N
COL5A1	hsa-miR-182	1	25.2564	57.9578	1.19835	1T
COL5A1	hsa-miR-9	1	25.2564	57.9578	1.19835	1T
AATK	hsa-miR-182	1	15.6319	2.48414	−2.65367	1T
AATK	hsa-miR-503	1	15.6319	2.48414	−2.65367	1T
AATK	hsa-miR-9	1	15.6319	2.48414	−2.65367	1T
GAB2	hsa-miR-486-5p	−1	17.3317	8.056	−1.10527	1N
GAB2	hsa-miR-9	1	17.3317	8.056	−1.10527	1N
DOT1L	hsa-let-7a	−1	14.0112	6.96755	−1.00786	1N1T
DOT1L	hsa-miR-200b	1	14.0112	6.96755	−1.00786	1N1T
DOT1L	hsa-miR-224	1	14.0112	6.96755	−1.00786	1N1T
DOT1L	hsa-miR-9	1	14.0112	6.96755	−1.00786	1N1T
THBS2	hsa-miR-182	1	10.5524	64.5186	2.61214	1N1T
THBS2	hsa-miR-30a	−1	10.5524	64.5186	2.61214	1N1T
THBS2	hsa-miR-9	1	10.5524	64.5186	2.61214	1N1T

N, normal; T, tumor; −1, downregulated in tumor; 1, upregulated in tumor; FC, fold change.
